# A comparison of inverted and upright laser-activated titanium nitride micropyramids for intracellular delivery

**DOI:** 10.1038/s41598-018-33885-y

**Published:** 2018-10-22

**Authors:** Alexander Raun, Nabiha Saklayen, Christine Zgrabik, Weilu Shen, Marinna Madrid, Marinus Huber, Evelyn Hu, Eric Mazur

**Affiliations:** 1 John A. Paulson School of Engineering and Applied Sciences, Harvard University, Cambridge, MA 02138 USA; 2 Department of Physics, Harvard University, Cambridge, MA 02138 USA; 3 Department of Physics, Ludwig Maximilian University of Munich, 80539 Munich, Germany; 4 Present Address: Johns Hopkins University Applied Physics Laboratory, Laurel, MD 20723 USA

## Abstract

The delivery of biomolecules into cells relies on porating the plasma membrane to allow exterior molecules to enter the cell via diffusion. Various established delivery methods, including electroporation and viral techniques, come with drawbacks such as low viability or immunotoxicity, respectively. An optics-based delivery method that uses laser pulses to excite plasmonic titanium nitride (TiN) micropyramids presents an opportunity to overcome these shortcomings. This laser excitation generates localized nano-scale heating effects and bubbles, which produce transient pores in the cell membrane for payload entry. TiN is a promising plasmonic material due to its high hardness and thermal stability. In this study, two designs of TiN micropyramid arrays are constructed and tested. These designs include inverted and upright pyramid structures, each coated with a 50-nm layer of TiN. Simulation software shows that the inverted and upright designs reach temperatures of 875 °C and 307 °C, respectively, upon laser irradiation. Collectively, experimental results show that these reusable designs achieve maximum cell poration efficiency greater than 80% and viability greater than 90% when delivering calcein dye to target cells. Overall, we demonstrate that TiN microstructures are strong candidates for future use in biomedical devices for intracellular delivery and regenerative medicine.

## Introduction

Intracellular delivery is a critical step in a variety of cell therapies, including cancer gene therapy and anti-HIV treatments^1–6^. Cargoes such as genetic molecules or proteins are delivered into the cytosol to alter the expressed traits of cells with powerful therapeutic implications. For example, small interfering RNA (siRNA) is delivered to cells to knock down the expression of genes associated with cardiovascular inflammation and central nervous system diseases^7–12^. Therapeutic genes are delivered to influence or replace faulty genes to treat immunodeficiency syndromes. Intracellular delivery of CRISPR-Cas9, a gene-editing tool, is being used to treat blood and eye diseases in humans^13–15^.

Due to the enormous impact associated with these cell therapies, there is a significant need for an effective platform that can deliver versatile cargoes to different cell types. While many biological, chemical, and physical intracellular delivery platforms exist, none combine high efficiency, high viability, high throughput, and low toxicity across a variety of cell lines and delivery payloads^16^. The most popular, established biological intracellular delivery method uses viral vectors to carry genes in a viral envelope and then inject them into the target cells^17–20^. While this technique has been optimized for years, it still has major disadvantages, such as frequent rejection of the vector by the immune system^21^. Physical *in vitro* delivery methods such as electroporation are well-established^22–24^. Despite electroporation’s high efficiency and throughput, the viability of the technique is low^25^. Plasmonic nanoparticles have also been used to perforate cell membranes. In this process, gold nanoparticles, which are in close contact with the target cell membrane, absorb energy from pulsed laser light, leading to super-heating and bubble formation in the solution surrounding the target cells; these bubbles induce membrane poration, allowing the desired payload to diffuse into the cells^26–29^. However, the gold nanoparticles often remain in the target cells after the treatment, leading to potential toxicity in the cells^30–32^.

A strong alternative to these methods is substrate-based delivery. This methodology can be traced back to pioneering studies using immobilized gold nanoparticles and metallic films on top of substrates such as glass and silicon^33,34^. More recently, a very promising, novel intracellular delivery platform uses structured, thermoplasmonic substrates^35–38^. These substrates are patterned with an array of gold, pyramid-shaped microstructures. As with the gold nanoparticle method, the pyramids locally absorb the laser energy, leading to hotspot formation at the pyramid apexes. Subsequent bubble formation in the surrounding solution opens the cells’ membranes for payload to diffuse into the cells. This intracellular delivery platform has been optimized to achieve efficiencies up to 95%, a viability of 98%, and a throughput of 50,000 cells/min (with the option to scale up by changing certain parameters such as laser scanning speed and beam diameter).

Even though gold pyramid substrates achieve the core goals of delivery efficiency, viability, and throughput, gold is a weak metal mechanically. Specifically, on the Mohs hardness scale, which measures the scratch resistance of materials, gold has a low rating of 2.5 out of 10^39^. We see this in substrate fabrication and handling, for the gold film easily scratches off of the underlying substrate. For long-term clinical applications, the weak mechanical properties of gold are not ideal; they could present issues in residual toxicity in cells and faster degradation over time^40^. In order to overcome this issue, this paper presents the replacement of gold as the active plasmonic material in this intracellular delivery platform with titanium nitride (TiN). TiN is an extremely robust material often used in industrial applications for strong protective coatings^41^. Specifically, TiN has a Mohs rating of 9 and a high melting temperature of 2947 °C, much higher than gold’s melting temperature of 1064 °C^42,43^.

In addition to TiN’s attractive mechanical and thermal qualities, it has garnered interest because of its unique plasmonic characteristics^44^. While TiN is traditionally thought of as a ceramic material, its non-stoichiometric composition allows its electronic behavior to be tuned from insulating (characteristic of a dielectric) to metallic (like gold and silver)^45^. This tuning can be done by changing critical synthesis parameters of TiN thin films, such as temperature during deposition, the underlying substrate, and type of deposition method (reactive *vs*. non-reactive sputtering)^45^.

This tunable electronic property is valuable for a thermoplasmonic application, for it will allow TiN to generate the hotspots necessary for bubble formation. This property has already been used to construct plasmonic TiN nanostructures, such as films, rods, wires, and nanoparticles, which show highly efficient, local heat generation in various wavelength ranges^46,47^. Finally, TiN is a biocompatible material, making it a safe material to use in intracellular delivery applications^48^.

We present thermoplasmonic substrates patterned with inverted and upright micropyramids with a thin TiN coating for intracellular delivery applications. We explored two designs, inverted and upright pyramids, in order to discover potential optimization opportunities. Additionally, we specifically chose geometrically opposite designs due to an inherent fabrication opportunity involving the process of template stripping (see Fabrication section). Both designs were fabricated and analyzed via scanning electron microscopy, and we carried out COMSOL simulations to determine critical experimental parameters and to understand nanoscale heating patterns. Finally, we conducted repeated substrate-use experiments to see if the delivery efficiencies and viabilities changed after substrates underwent repeated laser illumination.

## Results and Discussion

### Laser irradiation of cells on each substrate

**Figure 1:**
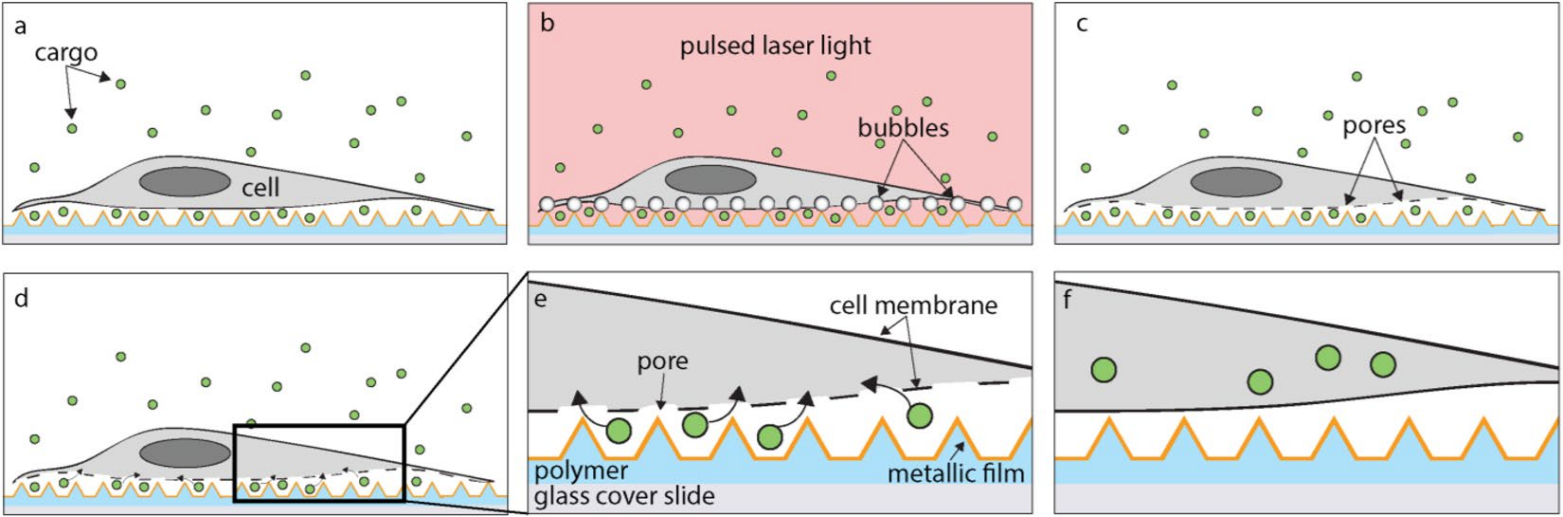
A schematic representation of a 2D cross-section of intracellular delivery using a thermoplamonic substrate in an aqueous environment. (**a**) A cell seeded onto metallic micropyramids surrounded by a solution containing delivery payload. (**b**) The whole system is irradiated with a large-area pulsed laser beam from above, resulting in the localized formation of explosive boiling and bubbles at each pyramid. (**c**) The bubbles induce pores in the cell membrane. (**d**) Delivery payload enters the cell through the pores. (**e**) An inset from (**d**) shows payload entering the cell. The composition of the pyramids is also shown, which include a polymer substrate and thin metallic coating. (**f**) The cell membrane heals and the payload is retained in the cytosol.

We illuminate thermoplasmonic substrates consisting of TiN pyramid arrays and cells that have adhered to those structures with pulsed laser light (Fig. 1). Before laser illumination, the target cells are submerged in an aqueous solution containing the delivery payload. Laser irradiation generates a hotspot at each microstructure, which is hypothesized to result in a rapid temperature increase and subsequent bubble formation within the solution. The bubbles are expected to mechanically open the cell membrane at each pyramid, creating pores through which delivery payload can enter into the cell. In Fig. 1, the pores are shown in the lower membrane of the cell closest to the substrate, allowing the payload to enter from the space between the cells and the pyramid array. However, if the cells are stretched very thin, the pores could potentially form on the upper membrane as well, allowing payload to enter from above. After laser illumination, the cell membranes heal themselves to successfully encapsulate the payload within the cell. The laser beam spot is large (1.2 mm diameter), making it possible to treat thousands of cells simultaneously. The beam spot is scanned across the entire substrate to enable the treatment of millions of cells in minutes.

### Fabrication

**Figure 2:**
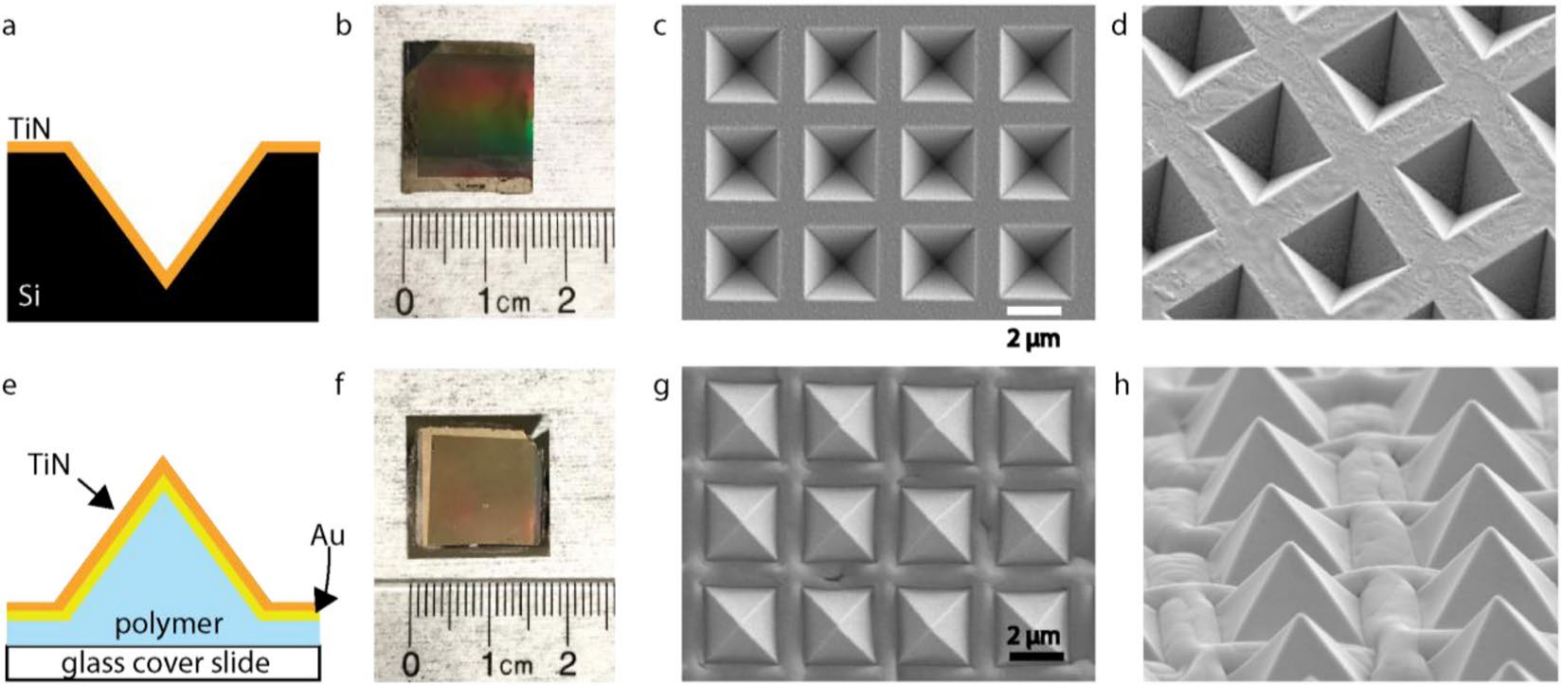
Inverted and upright TiN micropyramids. (**a**) A schematic of the cross section of the inverted pyramid design. (**b**) The inverted pyramid substrate next to a ruler. (**c**) Top-down view of the inverted pyramids with SEM. (**d**) Tilted SEM of the inverted pyramids, showing consistent fabrication dimensions for each structure. (**e**) A schematic of the cross section of the upright pyramid design. (**f**) The upright pyramid substrate next to a ruler. (**g**) Top-down view of the upright pyramid design taken via SEM. (**h**) Tilted view of the inverted pyramid design taken via SEM. This shows a consistent rising of the film in between each pyramid.

For the inverted pyramids, we sputter TiN directly onto an inverted pyramid silicon template, which is fabricated via anisotropic etching (Fig. 2a). For the upright pyramids, we use a combination of template stripping and sputtering to create polymer pyramids with a layer of gold and TiN on top (Fig. 2e). (The details of fabrication can be found in the Supplementary Materials, Fig. S1). Despite gold’s weaker attributes, the layer is necessary for the upright design’s template stripping process (see Methods Section)^49^. Additionally, because the gold is covered in TiN, it is protected from direct, incident laser light. Our fabrication processes for both designs result in rectangular substrates with side lengths on the order of centimeters (Fig. 2b,f).

Scanning electron microscopy shows uniform arrays of inverted pyramids and upright pyramids for both designs, respectively (Fig. 2c,g). The silicon inverted pyramids with a TiN layer on top shows uniform, high-quality fabrication (Fig. 2c,d). The SEMs of the multilayer, upright pyramids also show consistent fabrication of the pyramid structures (Fig. 2g). However, between the upright pyramids, a rising, or buckling of the film is present (Fig. 2h). In order to further understand this phenomenon, we etched the upright pyramids with a focused ion beam and then analyzed them with scanning electron microscopy (Fig. S2). The cause of this deformation requires further investigation, but it is most likely a surface wrinkling byproduct of the sputtering process^50,51^.

### Simulations

**Figure 3:**
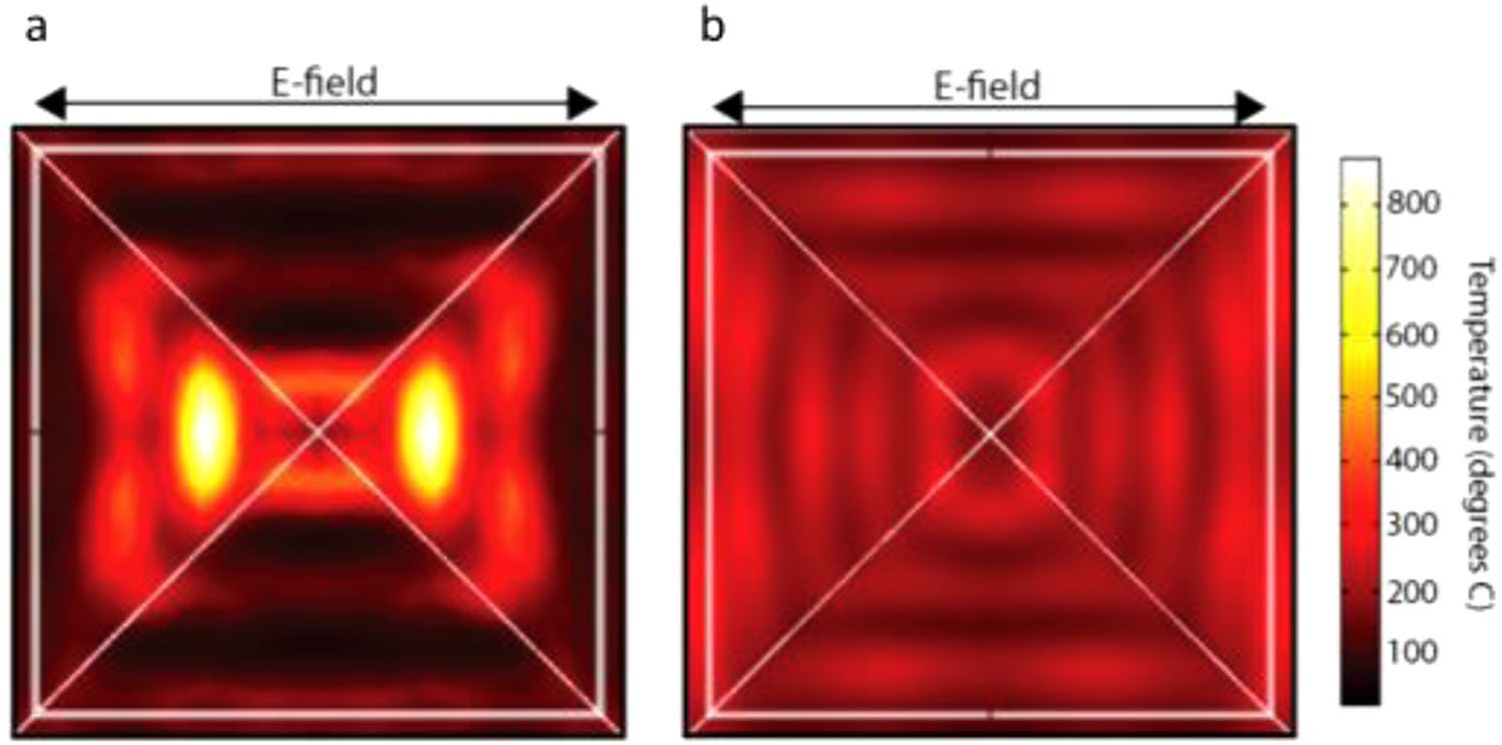
COMSOL temperature simulations. (**a**) A top-down view of an inverted pyramid, and the temperature distribution on the inclined surface of the structure when irradiated with a laser from the top with a fluence of 15 mJ/cm^2^. This shows the temperature of the 50-nm TiN film. (**b**) A top-down view of an upright pyramid, and the temperature distribution on the inclined surface of the structure when irradiated with a laser from the top with a fluence of 15 mJ/cm^2^. This shows the temperature of the 50-nm TiN film. Both simulations reflect actual pyramid sizes, with a base length of 2.4 µm and depth/height of 1.6 µm.

We explored the temperature distributions on both pyramid designs after laser irradiation using COMSOL (Fig. 3). To determine the bubble generation threshold, we studied irradiation at various laser fluences (Fig. S3). Figure S3A reveals that a laser fluence of at least 5 mJ/cm^2^ is necessary for explosive bubble formation on the inverted pyramids. The results from the upright pyramids in Figure S3B show that the bubble formation threshold is reached at 15 mJ/cm^2^. This explosive boiling can only occur at a temperature at or above 293 °C, based on thermodynamic principles and experiments^52^. For cell poration and viability experiments with both designs, we used laser fluences exceeding these thresholds, but below the damage threshold of the structures.

Figure 3 shows the temperature profiles of individual inverted and upright pyramids upon laser irradiation with a fluence of 15 mJ/cm^2^. In both simulations, the laser irradiates the structures from the top, and the images are top-down views. For the inverted structure, the simulation shows the maximum temperature is 875 °C after laser irradiation at a fluence of 15 mJ/cm^2^. The simulation shows a triangular pattern of hotspots on each side of the structure (corresponding to the light polarization, as indicated by the electric-field arrows). The inverted structure exhibits a significantly higher temperature profile. Both designs show similar spectrophotometry results (Fig. S4) at the 1064-nm wavelength used. However, the underlying silicon should theoretically absorb more light at this wavelength than the polymer, based on its absorption coefficient and the polymer’s high transmission level^53^. Overall, further understanding of the heat distribution and the thermal conductivities of the materials will be needed to confirm these results.

For the upright pyramids, the model shows that the hot spots form near the base of the pyramid structures, and reach a maximum temperature of 307 °C (Fig. 3b). When an upright pyramid-shaped structure is irradiated with light from underneath or above, a hotspot near the tip of the pyramid is expected, as is seen in the gold pyramid design^35^. However, the hotspot formation at the base of the pyramid for the upright pyramids is most likely due to the lower thermal conductivity of the TiN layer compared to gold. Because of this, the incoming light energy collected near the base of the pyramid cannot dissipate to the tip of the structure.

Laser ablation at fluences higher than the damage thresholds of both designs was carried out in order to attempt confirmation of simulation results (Fig. S5). For the inverted structures, the damage pattern confirms the COMSOL simulation (Fig. S5A). When the upright pyramids are ablated at a laser fluence above the damage threshold, there is a consistent damage pattern on the right side of the pyramids near the base (Fig. S5B). This reflects the initial COMSOL results. It is not clear why damage appears only on the right side of the pyramids. Based on the fabrication results of the upright pyramids, more hotspot generation could occur between the structures due to the rising of the film, further contributing to the damage pattern near the base of the pyramids seen under SEM. Additionally, the underlying gold film could be melted underneath the TiN due to its lower melting temperature^43^.

### Poration efficiency and viability

**Figure 4:**
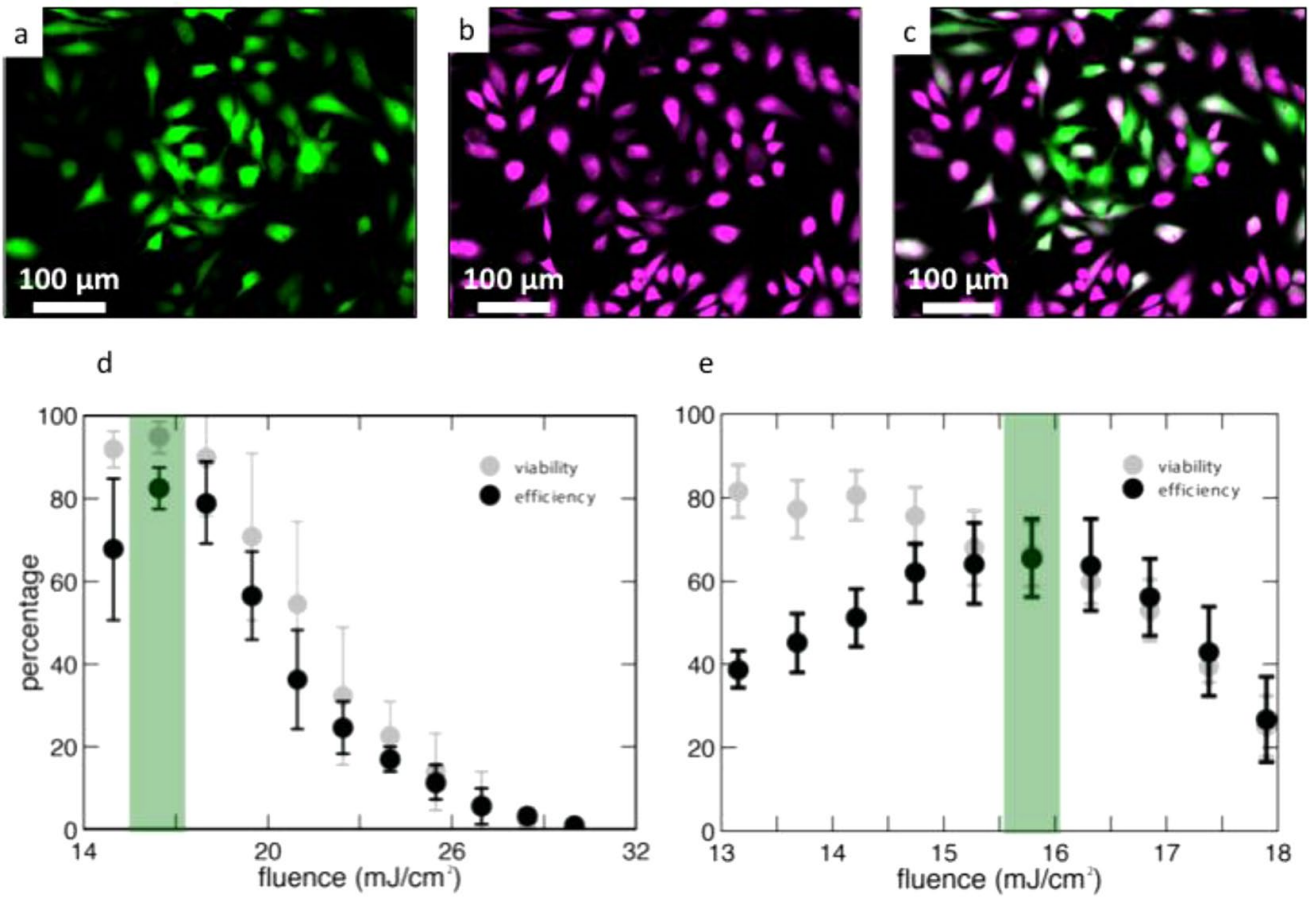
Cell poration efficiency and viability results. (**a**–**c**) represent an example of cell counting methodology (**a**) Green cells represent cells that were porated and uptook calcein Green dye. (**b**) The same area of cells showing the cells that survived. (**c**) An overlay of (**a**) and (**b**). (**d**) Quantified efficiency and viability results for the inverted pyramid substrate. The green bar highlights the optimal fluence for maximum efficiency and viability. (**e**) Quantified efficiency and viability results for the upright pyramids. The green bar highlights the optimal fluence for maximum efficiency and viability. Error bars represent +/−SEM with *n* = 3.

We quantified the efficiency and viability after delivering calcein green to HeLa cervical cancer cells via laser irradiation using fluorescent microscopy (Fig. 4a–c). The wavelength used in experiments was 1064-nm. Inverted pyramids achieve a maximum poration efficiency of 83% and a viability of 95% at a laser fluence of 16.5 mJ/cm^2^ (Fig. 4d). As the laser fluence decreases/increases below/above 16.5 mJ/cm^2^, there is a drop in poration efficiency, and viability decreases with increasing laser fluence. For the upright pyramids, the maximum poration efficiency of 66% occurs at a laser fluence of 15.8 mJ/cm^2^ (Fig. 4e). At this fluence, the viability is 67%. Viability maximizes at 82% at 13 mJ/cm^2^ and decreases as the laser fluence increases. As described in the materials and methods section, a green cell is counted as porated, and a magenta cell is counted as viable, regardless of the fluorescence gradient within the cell. This is to make the cell counting process binary in nature. The cell counting process for efficiency is valid because every green cell (indicating delivery) in Fig. 4a also shows up in the magenta channel (indicating viability). We confirm this in Supplementary Fig. S9. Additionally, through this initial evaluation of device performance in Fig. 4d,e, we highlight in green the maximum efficiency achieved and at what viability this occurred.

The trends in both data sets are expected. At lower fluences, more cells survive because there is a smaller disruptive effect on their membranes. As fluence increases, the membranes are disrupted more, leading to an increase in cell death. With respect to efficiency, at low fluences, the cell membranes do not open, leading to low efficiency values. As fluence increases, the membranes become more permeable, leading to greater molecular uptake. After the fluence increases beyond the optimum fluence, efficiency drops, as calcein leaves the dead cells through their highly porous, leaky membranes, a phenomenon seen in other intracellular studies^34,38^. As all porated cells are viable, the viability and poration efficiency curves match each other with increasing laser fluence. Overall, both designs show encouraging results in efficiency and poration. The inverted pyramids’ maximum efficiency of 83% and viability of 95% are comparable to the values achieved through the fully optimized gold pyramid platform^35^. Finally, higher temperatures simulated with the inverted structures in Fig. 3a do not kill more cells than the upright structures, because the hotspots are highly localized, both spatially and temporally. Additionally, this phenomenon could be due to the hotspots forming farther away from the cell membrane, since they occur at the tip of the inverted shape. Alternatively, additional, weaker hotspots could be forming near the base of the inverted structures at lower temperatures^54^.

### Repeated Use

**Figure 5:**
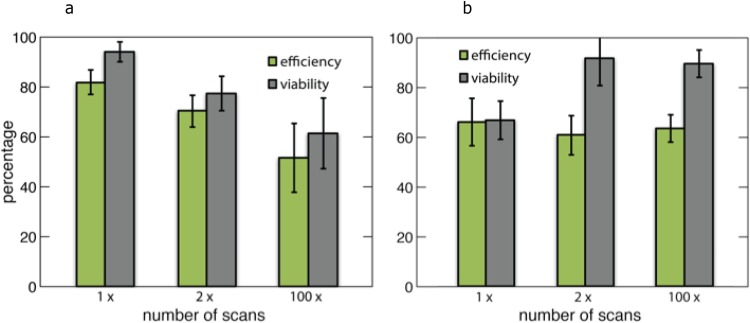
Maximum poration efficiency and viability of the designs after repeated laser illumination. (**a**) Maximum efficiency and viability values for inverted pyramids after 1, 2, and 100 scans. (**b**) Maximum efficiency and viability values for upright pyramids after 1, 2, and 100 scans. In these figures, the error bars represent +/−SEM and n = 3. p-values for all pairs are greater than 0.05 with an ANOVA test.

In order to determine the reusability of the substrates, we studied the poration efficiency after 1, 2, and 100 laser scans of the two substrates (Fig. 5).

The inverted pyramids exhibit decreases in both efficiency and viability as the number of scans increases from 1 to 100. However, this trend is not statistically significant (*p* > 0.05). Additionally, scanning electron microscopy reveals that substrates degrade under repetitive laser scanning, but not in the defined laser damage pattern seen previously (Fig. S6A). This damage is most likely due to external factors, such as substrate handling and repeated submersion in aqueous solutions.

The upright pyramids show consistent efficiency performance all the way up to 100 scans of reuse. Additionally, under the scanning electron microscope, this design shows no damage in any repeated scan range (Fig. S6B).

## Conclusion

We fabricated and compared two designs of TiN micropyramid arrays. We analyzed these designs using COMSOL temperature simulations and scanning electron microscopy after fabrication and laser irradiation. Both designs were used to deliver calcein dye to HeLa cervical cancer cells. The designs’ performances were analyzed after multiple laser irradiations to test reusability. The inverted pyramid structures in silicon with a film of TiN achieve high poration efficiency and high viability levels of 83% and 95%, respectively. Both of these values are comparable to the values obtained with fully optimized gold pyramid structures, which are at 95% and 98%, respectively^35^. Additionally, the upright pyramids show no degradation in performance after extensive reuse, making them a strong candidate for a reusable substrate in intracellular delivery.

Because this platform can be optimized further, there is great promise for improvements in intracellular delivery via TiN micropyramid substrates. For the upright pyramids, optimization of the laser setup, fabrication steps, or structure geometry could improve the substrate’s efficiency and viability in treating cells. Further research is required to study the reusability of the inverted pyramids, due to a potential trend in decreasing performance with more reuses and visible damage under scanning electron microscopy. Additionally, understanding the interaction between different materials in an optimal geometrical design is crucial to achieving this goal, for material interface effects such as differential heat expansion could have significant impacts on device performance and sustainability over time. In addition to optimization, a pump probe setup could be used to measure temperatures reached during irradiation and to validate the pore-opening mechanism. For both designs, versatility can be explored by using different target cell types, like pancreatic cancer cells, suspension cells, and muscle cells. Additionally, more payload types could be explored for delivery. Specifically, the delivery of larger molecules that approach molecular weights on the order of thousands of kDa could be tested. In addition to dyes, functional *in vitro* experiments using cargo such as siRNA and functional proteins could be carried out. The results of intracellular delivery using TiN substrates with minimal optimization presented here, however, prove TiN’s strong potential as a new plasmonic material for intracellular delivery applications. Upon further optimization, TiN will make for strong substrates for long-term clinical use.

## Methods

### Silicon template fabrication

In order to fabricate both pyramid designs, a grid of inverted pyramid structures in a silicon wafer is created. To do this, Chromium is deposited onto the silicon wafer. Lithography is then used to etch away a grid of squares in the chromium layer (these squares correspond to the bases of the pyramids). Next, KOH is used to anisotropically etch inverted pyramids into the silicon through the squares in the chromium. The chromium layer is then lifted off, resulting in the final inverted pyramid template (Fig. S1.1–6). The detailed steps for this process can be found in Saklayen *et al*.^35^.

### Titanium nitride deposition on inverted pyramids

For the inverted pyramids, a 50-nm layer of TiN is sputtered onto the silicon template (Fig. S1.7A). This is done using a DCV magnetron sputtering system. 40 sccm of Argon gas is flowed through the sputtering chamber. The TiN target plasma is ignited at a pressure of 25 mT. After the ignition, the target power is ramped up to the maximum value of 50% of the total power, which corresponds to 150 W and 393 V. This ramp up is done at a pressure of 4 mT. After ramping up, an 8-W, 90-V bias is applied to the substrate. Immediately after, the shutter over the TiN target is opened, allowing the sputtering process to begin. Because this process takes place at room temperature, the 90-V substrate bias is necessary in order to ensure a metallic TiN layer is sputtered^45^. The other parameters are standard for sputtering any thin film in the sputtering system used, and they are also based on optimization studies for the fabrication of metallic TiN films.

### Fabrication of template stripped, upright pyramids

For the upright pyramids, 50 nm of gold is deposited via an electron-beam evaporator onto the silicon-inverted pyramids. A No. 1.5 glass coverslip is then glued to this template with UV-curable glue (Norland Adhesive 61) and cured under a UV lamp. The gold, cured polymer, and glass are peeled off from the template using a razor blade to produce the gold pyramid structures (Fig. S1.7B–10B). Finally, a 50-nm layer of TiN is sputtered onto the gold pyramids using the same sputtering parameters described above (Fig. S1.11B).

### COMSOL Temperature Simulations

The Finite Element Method (COMSOL Multiphysics, 4.4) simulations solve for the theoretical temperature reached on a single pyramid structure when irradiated with a pulsed laser from the top of the structure. The simulation built is based on a previous simulation developed for the gold pyramids^35^. This simulation includes water as the medium surrounding the structure. The laser parameters used in the simulation include a wavelength of 1064 nm, 11 ns pulses, and a 50-Hz repetition rate. These properties reflect the laser used for experiments. For the inverted structures, the geometrical parameters include a pyramid base length of 2.4 µm, a pyramid depth of 1.6 µm, a spacing of 1.2 µm, and a TiN thickness of 50 nm. For the upright pyramids, the same pyramid dimensions are used, and a 50-nm layer of gold is incorporated beneath the TiN layer. All of these characteristics were chosen to resemble the actual fabricated samples. The simulation takes the power loss/energy absorbed from a linearly polarized, Gaussian pulse of 11 ns when irradiating a pyramid and converts this into a heat source in the film. More details about the setup of the simulation can be found in Demésy *et al*.^55^.

The properties of water and gold are taken from Ekici *et al*.^56^. The thermal properties of TiN (thermal conductivity, heat capacity, density) are obtained from the COMSOL Multiphysics materials library. The refractive indices of TiN in both simulations at the light wavelength of 1064 nm are estimated using ellipsometry data from Zgrabik *et al*.^45^. Finally, the properties of the UV glue are provided by the manufacturer (Norland).

In order to determine which laser fluence would be an ideal value to observe poration, a parametric sweep of several laser fluences is conducted in this simulation. As was stated, a temperature of at least 293 °C is necessary for bubble formation and subsequent cell membrane poration. Therefore, laser fluences that provide this temperature profile would be valid options for experiments. Five laser fluences are simulated: 5,10,15,20, and 25 mJ/cm^2^. This range was determined based on experimental data obtained from gold pyramid irradiation^35^.

### Seeding cells

Dye delivery experiments were conducted on the inverted and upright pyramids. For these experiments, a HeLa CCL-2 cell line is used. The cells are used at a passage number between 10 and 30 and cultured in a solution of DMEM containing 10% FBS and 1% penicillin streptomycin. The cells are incubated at 37 °C and 5% CO_2_. In order to seed the cells on the pyramid substrates, the cell media is removed from a T-75 flask using an aspirating pipette. 5 mL of PBS is used to wash the cells for 10 s. The PBS is then removed and the cells are covered with 3 mL of trypsin. After incubating the cells in the trypsin for 5 min, at least 80% cell detachment is observed using a microscope. 6 mL of media is added to the cells to neutralize the trypsin. This entire solution is then placed in a 15 mL tube and centrifuged for 5 min at 125 g. After the centrifuge step, the supernatant is gently removed from the cells with a pipette. The cells are then resuspended in 8 mL of media. Using a Countess cell counter, the cell density and viability are validated. Based on the concentration given by the cell counter, the cells are resuspended in cell media to obtain a cell concentration of 700,000 cells/mL. Next, 1.4 million cells, or 2 mL of the solution, are seeded on top of a substrate within a 35-mm petri dish. For triplicate experiments, three separate substrates in three separate 35-mm petri dishes are used. These cells are then incubated overnight in order to ensure cell attachment to the substrate.

### Laser scanning experiments

Each substrate with cells attached to the surface is removed from the culture petri dish and submerged in another 35-mm dish with 2 mL of PBS. 200 µL of calcein green dye (concentration = 0.57 mg/mL), a 0.6-kDa dye, is added to this volume of PBS. This dish with the substrate, cells, and green dye is placed on a microscope scanning stage. The laser is calibrated through a measurement of the beam spot size at the stage and a subsequent energy measurement at the stage. Based on the beam spot size and an energy measurement, the laser fluence is determined by dividing the laser energy delivered at the stage by the beam spot area.

In all experiments, the beam spot is ~1–1.2 mm in diameter. The laser power on the built-in laser attenuator for experiments is set to 90–100%. Additional laser attenuation tuning is done via a half-wave plate and polarizer connected to a widget on a computer. Once the range of laser fluences is calibrated with corresponding half-wave plate settings, the substrate is scanned at the laser fluences of interest. For each laser fluence, the scanning stage is set to a ∆*x* of 100,000 µm, a ∆*y* of 500 µm, and a scanning speed of 10,000 µm/second. The ∆*x* value is set to 100,000 µm to ensure a uniform speed in the center of its path where the substrate is irradiated with a laser. The ∆*y* value was set to 500 µm, and the laser is blocked on every other scan to ensure full separation between laser scans at different fluences. The scanning speed value of 10,000 µm/second is based on the optimal scanning speed developed for the gold pyramid platform.

The throughput of the system can be quantified with these parameters chosen. The samples, which are roughly 225 mm^2^, take up 20% of the 35 mm (diameter) petri dish area. Therefore, about 20% of the cells seeded in the dish are on the substrate (20% of 1.4 million = 280,000 cells). The number of cells per area is then (280,000 cells)/(225 mm^2^) = 1,244 cells/mm^2^.

In a single laser scan, the area treated can be defined as a rectangle with a width of 500 µm (∆y) and a length of 15 mm (the length of the substrate). The ∆y value of 500 µm is used, for when scanning the whole substrate at one fluence, the laser is unblocked during every pass to ensure full coverage of the substrate. For this 7.5 mm^2^ area of one laser scan, there are (1,244 cells/mm^2^) (7.5 mm^2^) = 9,330 cells.

With a ∆*x* of 100,000 µm and scanning speed of 10,000 µm/second, these cells were treated in 10 s, producing a rate of 933 treated cells/s. This translates to a throughput of ~56,000 cells/min, which matches the throughput of the gold pyramid platform^35^. Additionally, this set up could be changed by using scanning mirrors or by using a larger beam spot to scale up the throughput.

After laser scanning, the substrate with porated cells is transferred to a 35-mm petri dish with 2 mL of calcein AM red-orange viability dye. This dye only enters and fluoresces red in live cells. The cells are incubated in the viability dye for 15 min prior to imaging.

### Cell counting

To count poration efficiency and viability of the treated cells, a Nikon fluorescent microscope is used. For each laser fluence, an image is taken in the green and red fluorescence channels. We consider a cell to be porated or alive regardless of the internal fluorescence gradient. This converts each cell’s viability and poration efficiency to a binary value, which makes the counting process clear and repeatable. This counting approach is depicted in Fig. S9. In Fig. S9, we show the outlines of each viable cell from Fig. 4b and then overlay the outlines on top of the green, porated cells from Fig. 4a. Figure S9 confirms that every green porated cell from Fig. 4a is also viable. The cells are counted in these images using ImageJ and then compared to a control, an unscanned section of the substrate, in order to obtain a percentage for efficiency and viability. The control was defined in this way, for our optimized cell seeding process produces uniform cell density across the substrate. Additionally, by counting the control in this way, we ensure that the cells remain highly viable by only imaging them for a short period of time post-experiment. The percentages were calculated as follows:

$${Efficiency}\, \% =\frac{{Numbe}{{r}}_{{green}{cells}}}{{Numbe}{{r}}_{{control}}}\times 100$$

$$Viability\, \% =\frac{{Numbe}{{r}}_{redcells}}{{Numbe}{{r}}_{{control}}}\times 100$$

Finally, to produce Fig. 4a–c, the red color of viable cells is altered to magenta in ImageJ.

### Repeated Use Experiments

For repeated use experiments, substrates are scanned in a 35-mm petri dish in 2 mL of water without cells. Each substrate is divided into two sections. In one section, the substrate is scanned once, and in the other section, 99 times. For these repeated scans, the optimum poration efficiency fluence determined from cell counting is used.

The substrates are analyzed under a scanning electron microscope before testing the performance of the substrates. This is done by following the same seeding process described above. Both sections of the substrates are scanned uniformly at the optimum fluence value. The substrates are then submerged in the calcein AM red-orangeviability dye for fluorescent imaging and counting.

## Electronic supplementary material

Supplementary Figures
