# Supplementary material for: A comparison of inverted and upright laser-activated titanium nitride micropyramids for intracellular delivery
Source: Sci Rep. 2018 Oct 22;8:15595. doi: 10.1038/s41598-018-33885-y (PMC6197185; doi:10.1038/s41598-018-33885-y)
Supplement: Supplementary file 1 — Supplementary Figures [file 41598_2018_33885_MOESM1_ESM.docx]

Supporting Information for

**A comparison of inverted and upright laser activated titanium nitride micropyramids for intracellular delivery**

Alex Raun*, Nabiha Saklayen, Christine Zgrabik, Weilu Shen, Marinna Madrid, Marinus Huber, Evelyn Hu, Eric Mazur*

*Corresponding author. Email: raun.alex@gmail.com (A.R.); mazur@seas.harvard.edu (E.M.)

**This PDF File includes:**

Figures S1 to S9.


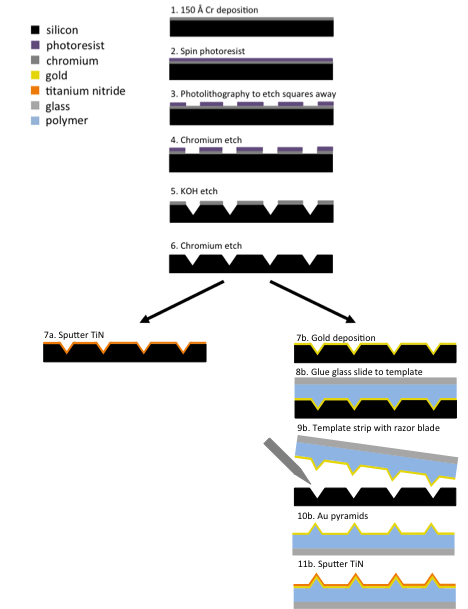


Figure S1. The fabrication steps for inverted and upright TiN pyramids.


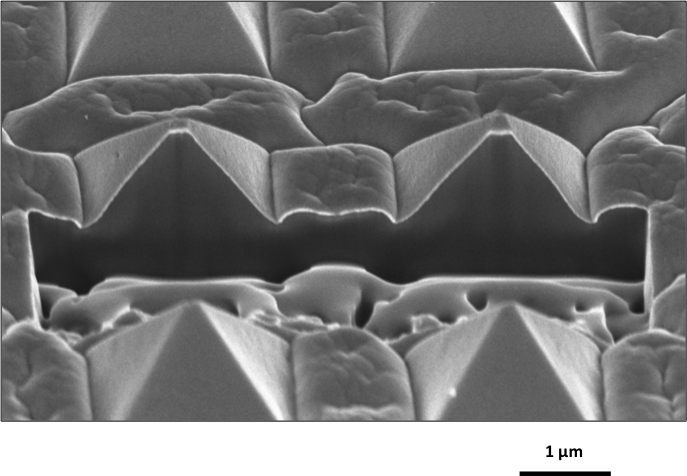


The gold-polymer interface

Figure S2. A cross-section of the upright pyramids created with FIB. This shows polymer present everywhere underneath the metallic film.

b

a


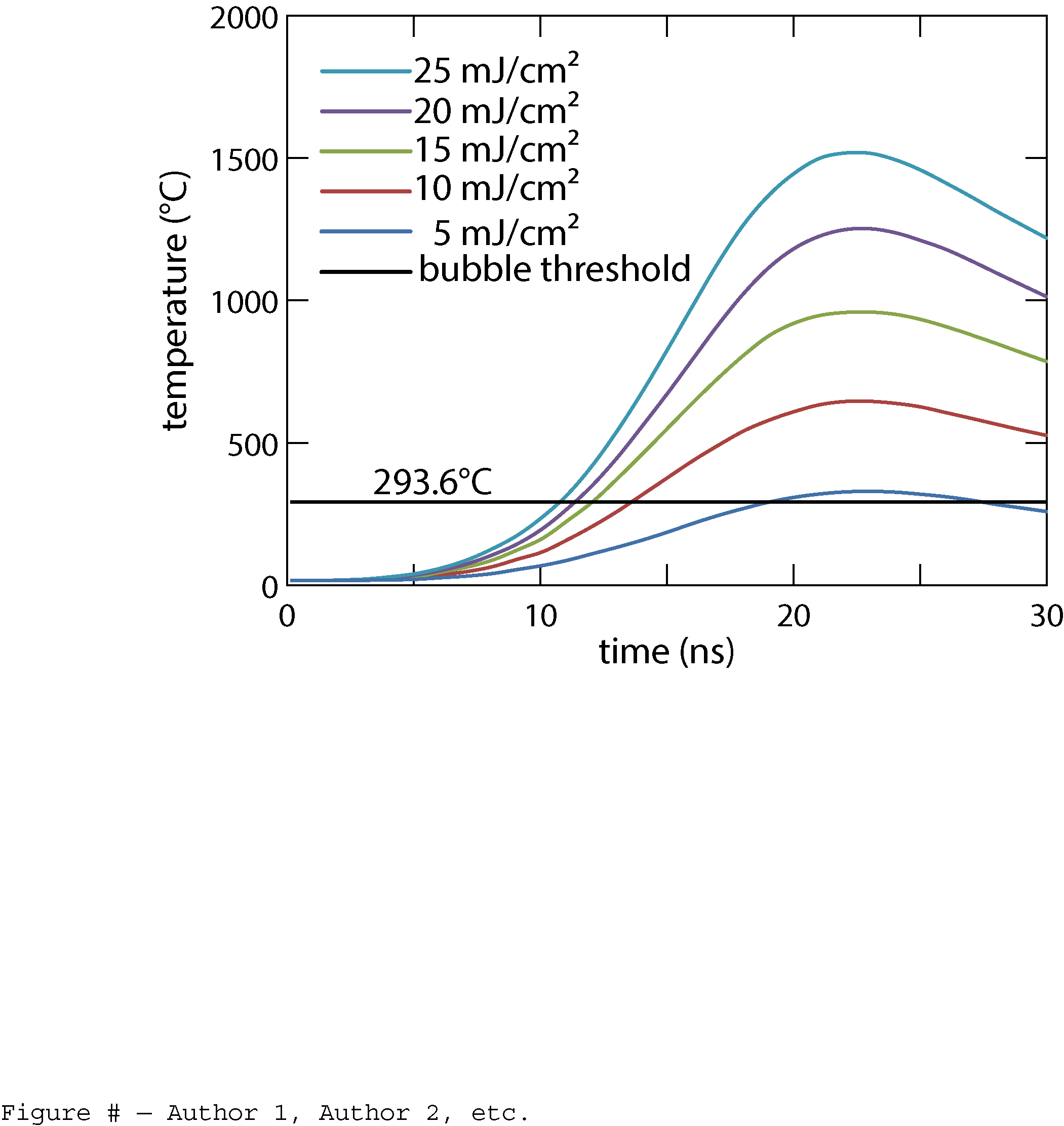

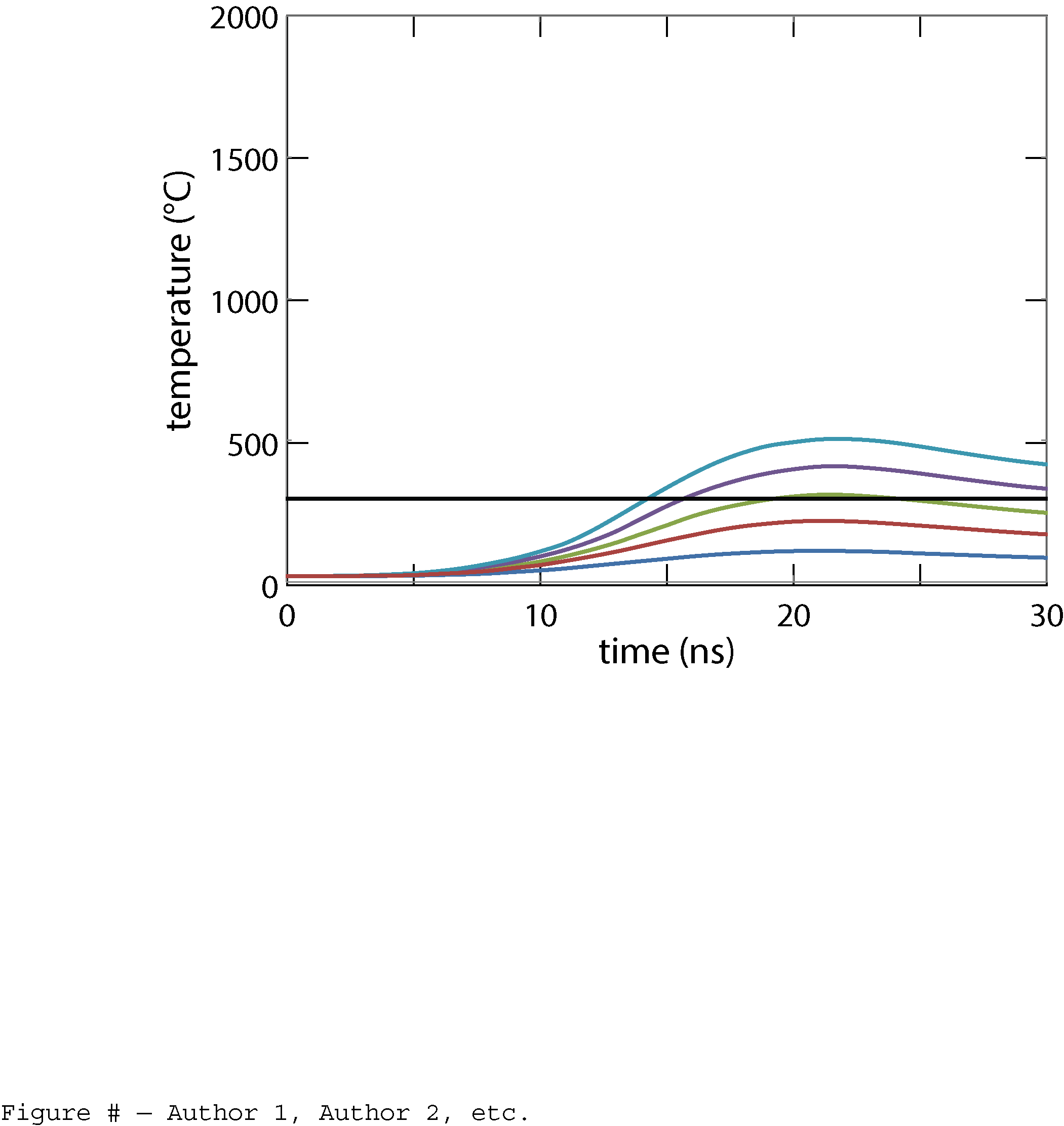


Figure S3. The maximum temperature (y axis) reached on a pyramid structure with respect to time (x axis) for different laser fluences. (A) The simulated temperature for the inverted pyramid design. (B) The simulated temperature for the upright pyramid design. The black line in both graphs represents the temperature necessary to create bubbles (293.6°C).


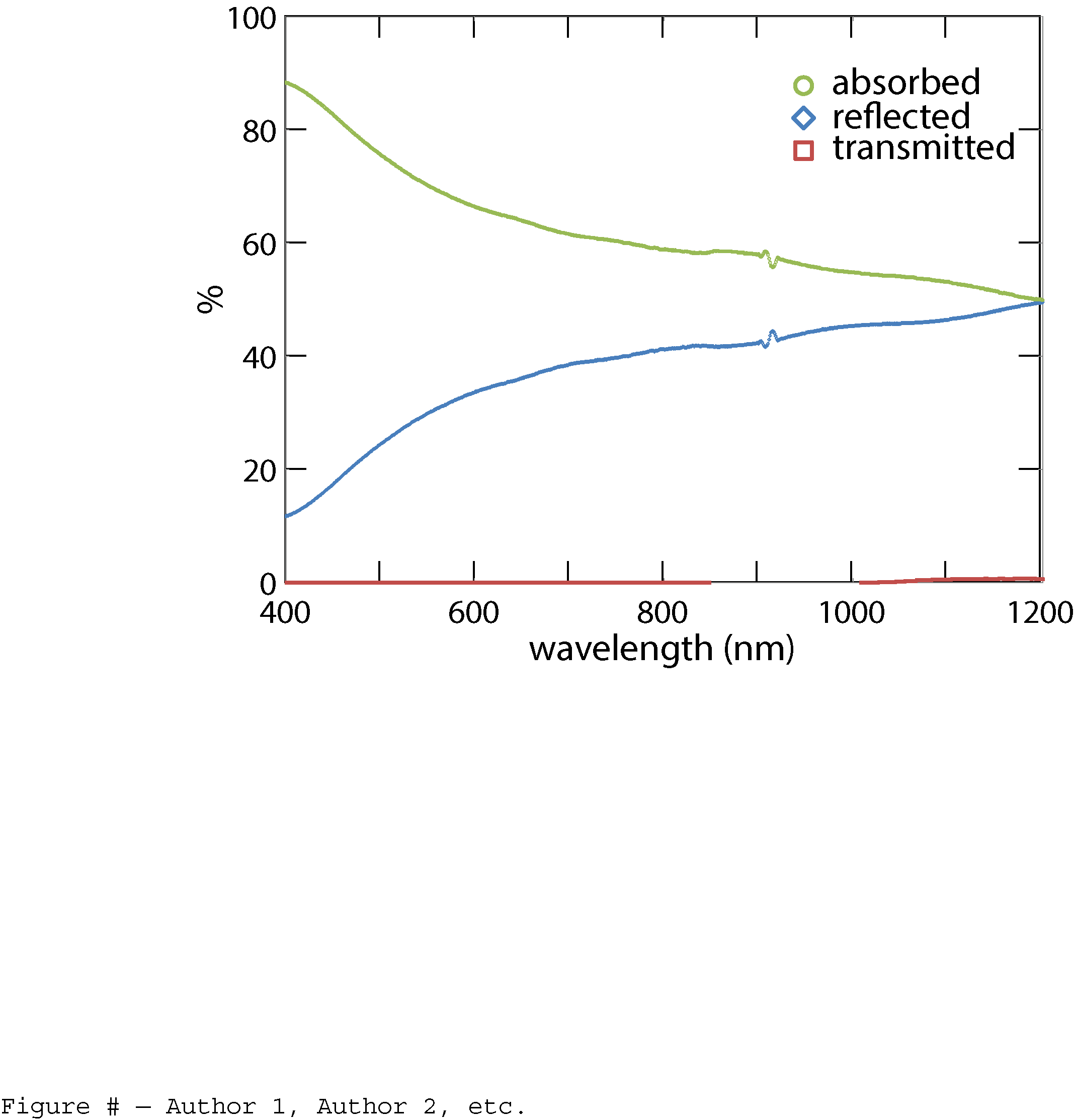

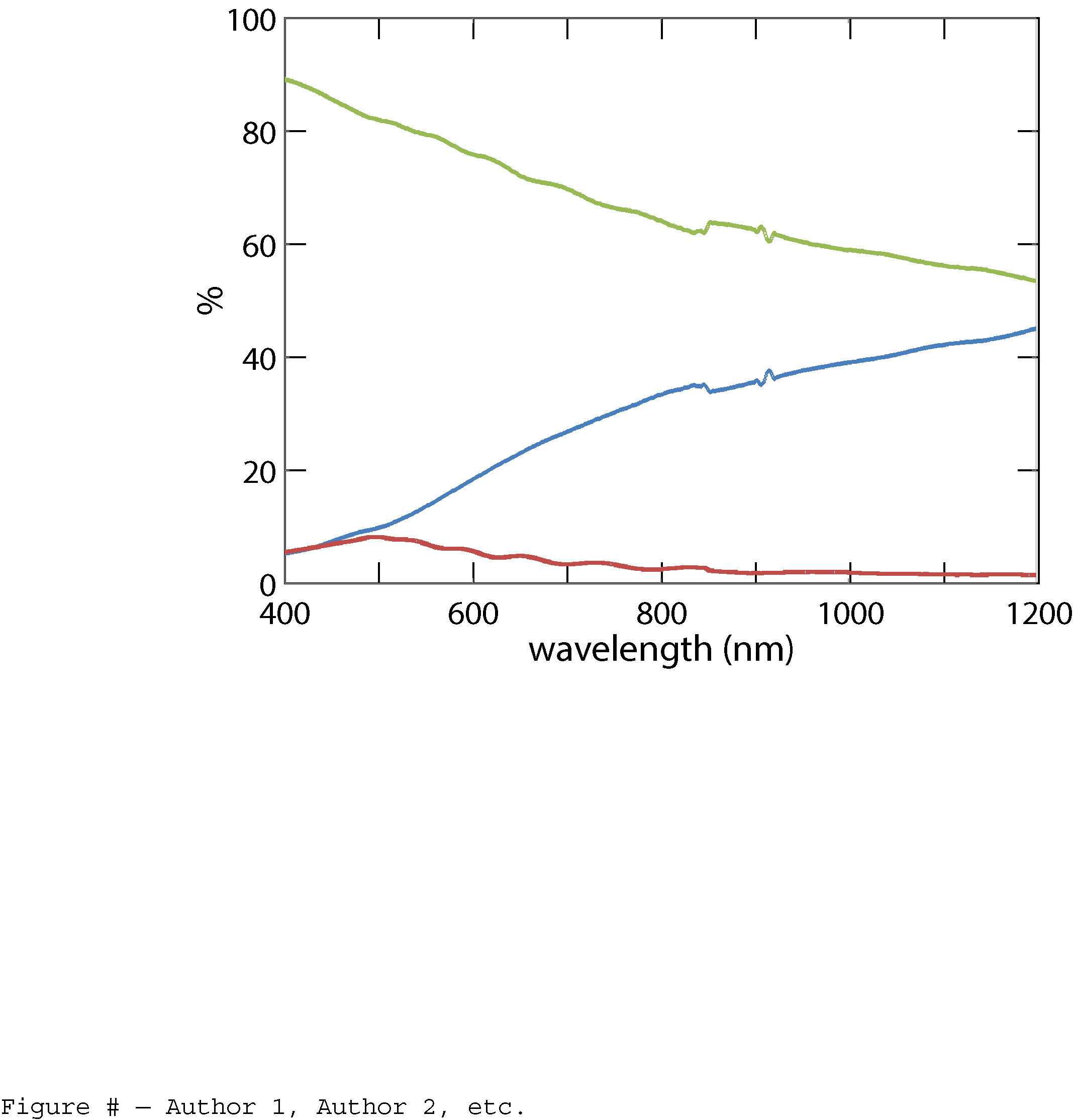


b

a

Figure S4. The spectrophotometry results for each pyramid design, showing the percent of light absorbed, reflected, and transmitted as a function of wavelength. (A) Results for the inverted pyramids. (B) Results for the upright pyramids.




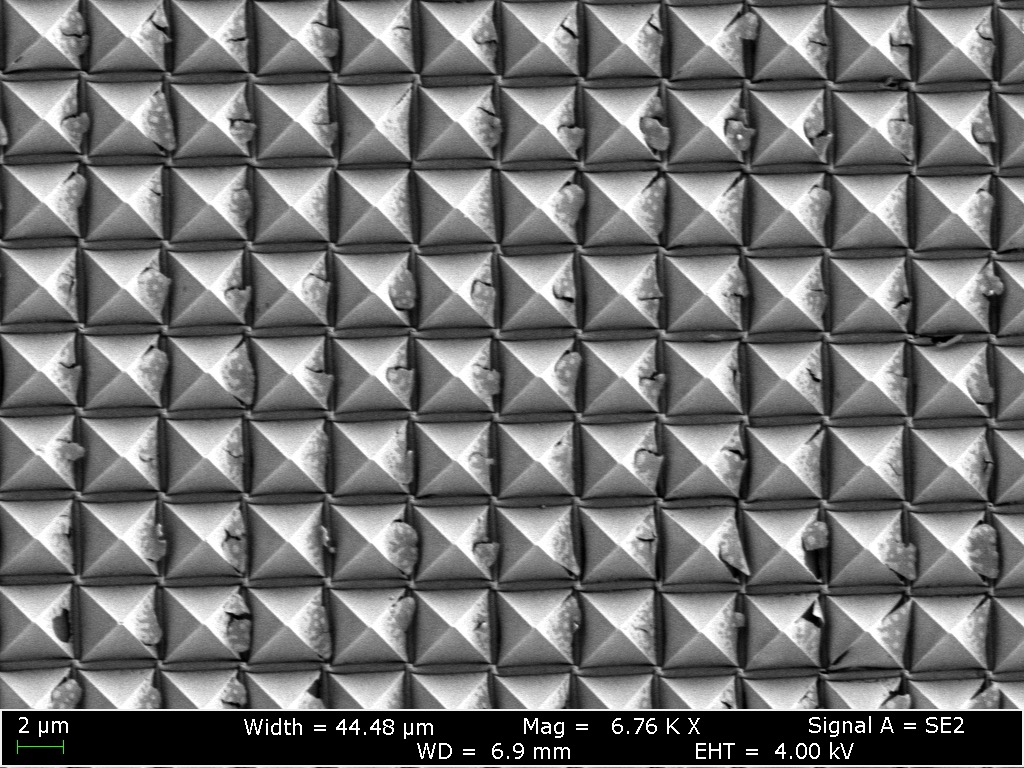


b

a

Figure S5. The damage patterns produced on both pyramid designs when ablated at a laser fluence significantly higher than the damage threshold of the structures. (A) Damaged inverted pyramids, showing a pattern consistent with the COMSOL simulation. (B) Damaged upright pyramids.


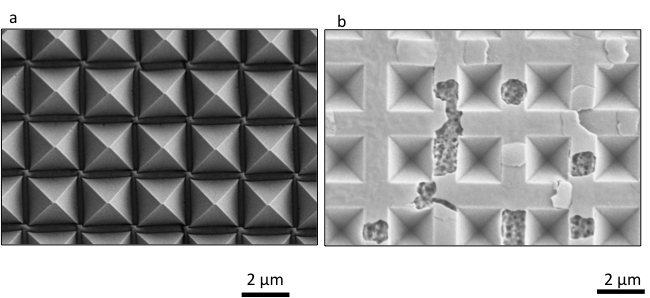

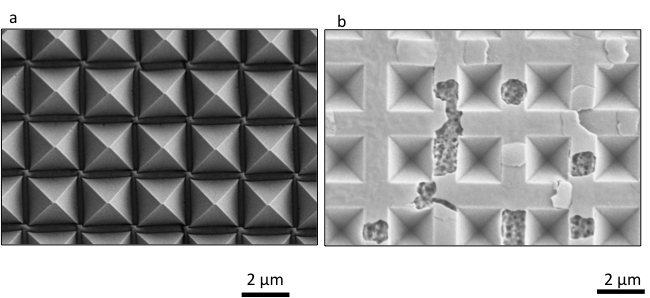


b

a

Figure S6. SEM of both designs after 100 laser irradiation scans. (A) These are inverted pyramids. Visible damage can be seen, however, this damage does not appear in the laser hot spot patterns, meaning this damage is due to external handling factors. (B) These are upright pyramids after 100 laser scans, and no visible damage on the structures can be seen.


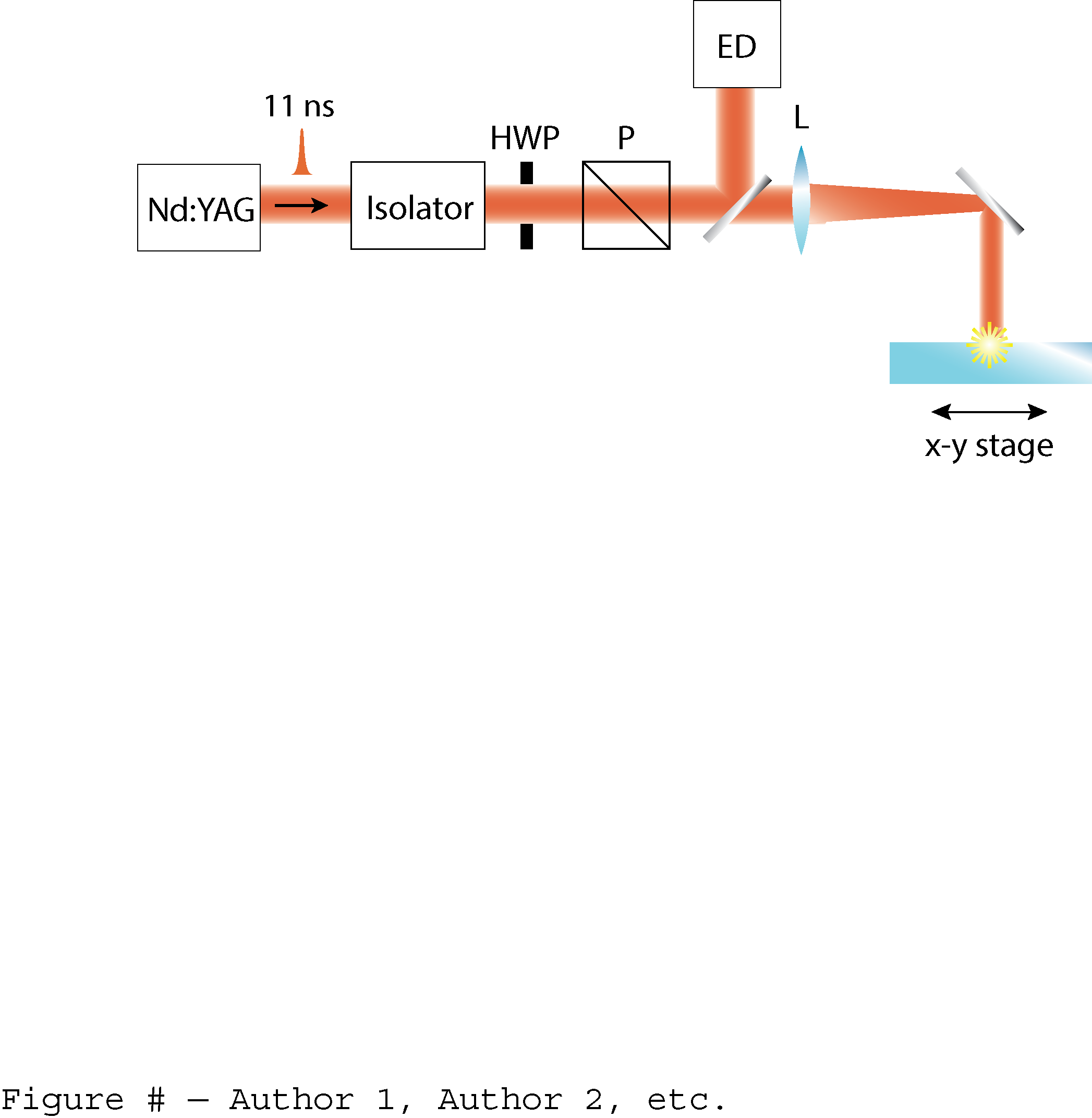


Figure S7. The laser setup used for intracellular delivery experiments. HWP = Half wave plate; P = Polarizer; ED = Energy detector; L = Lens





Figure S8. A HeLa cell chemically fixed to the inverted pyramids. The cell fixing procedure was taken directly from Ref. 33


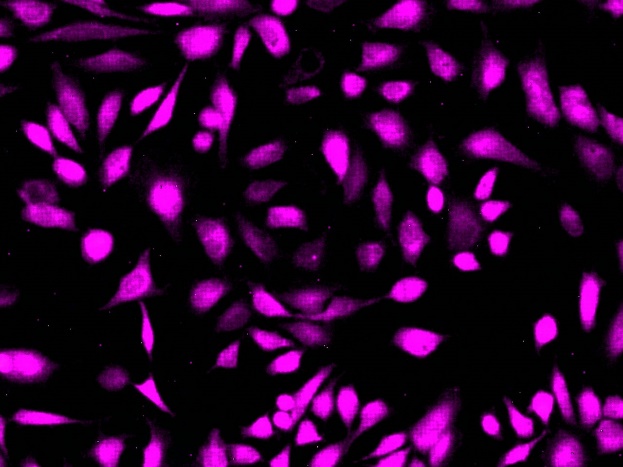


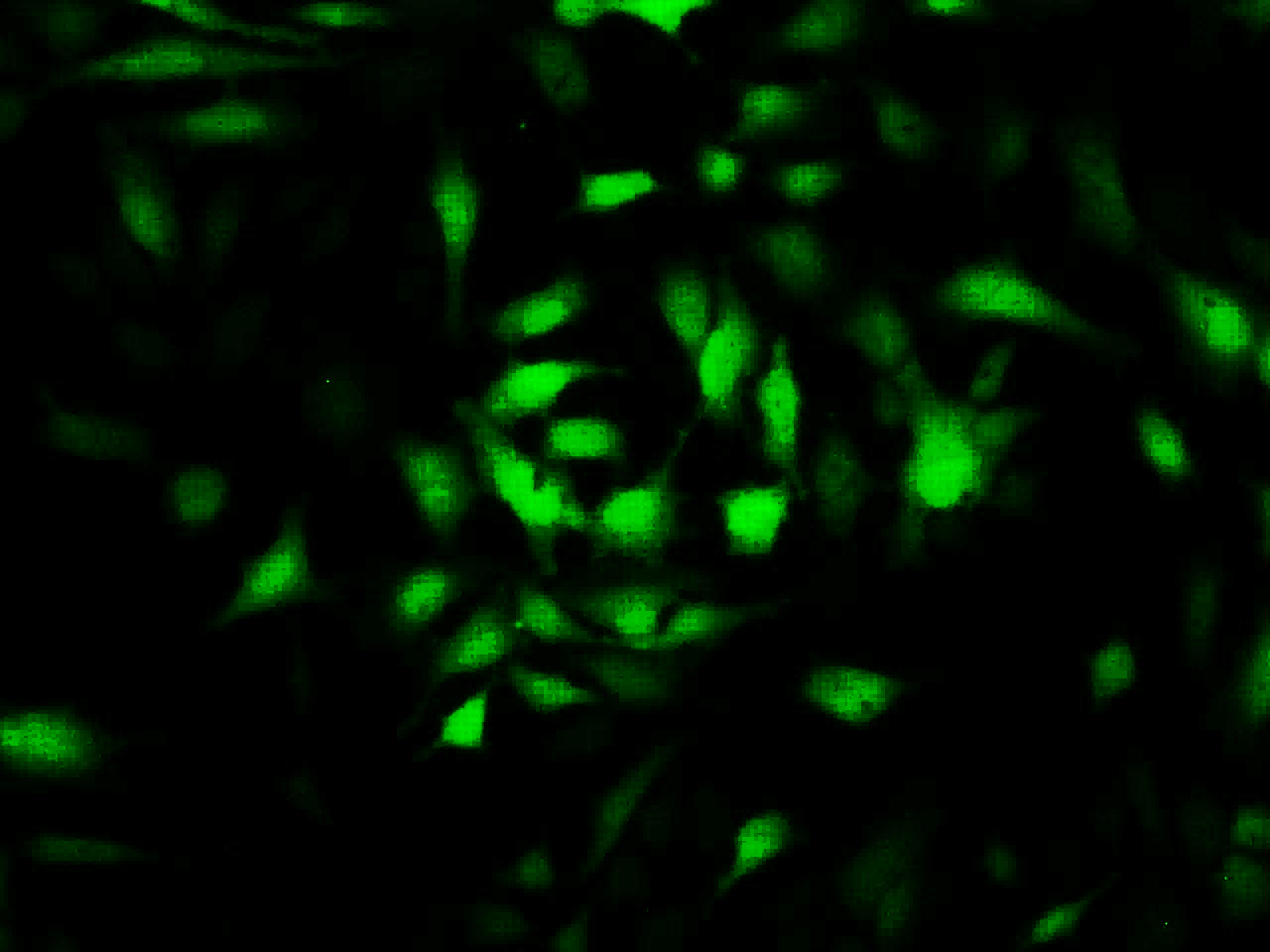

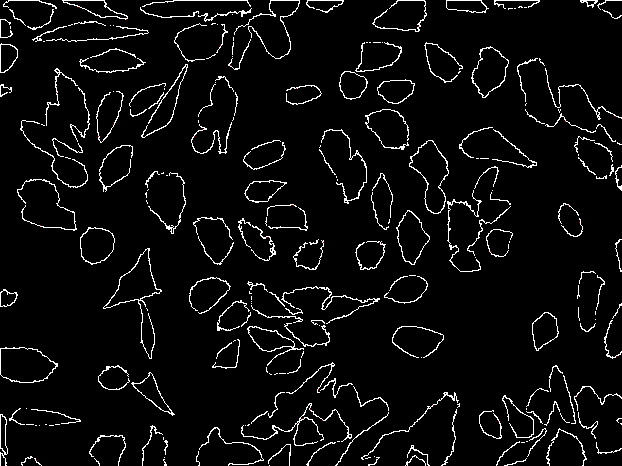


a

b

c

d


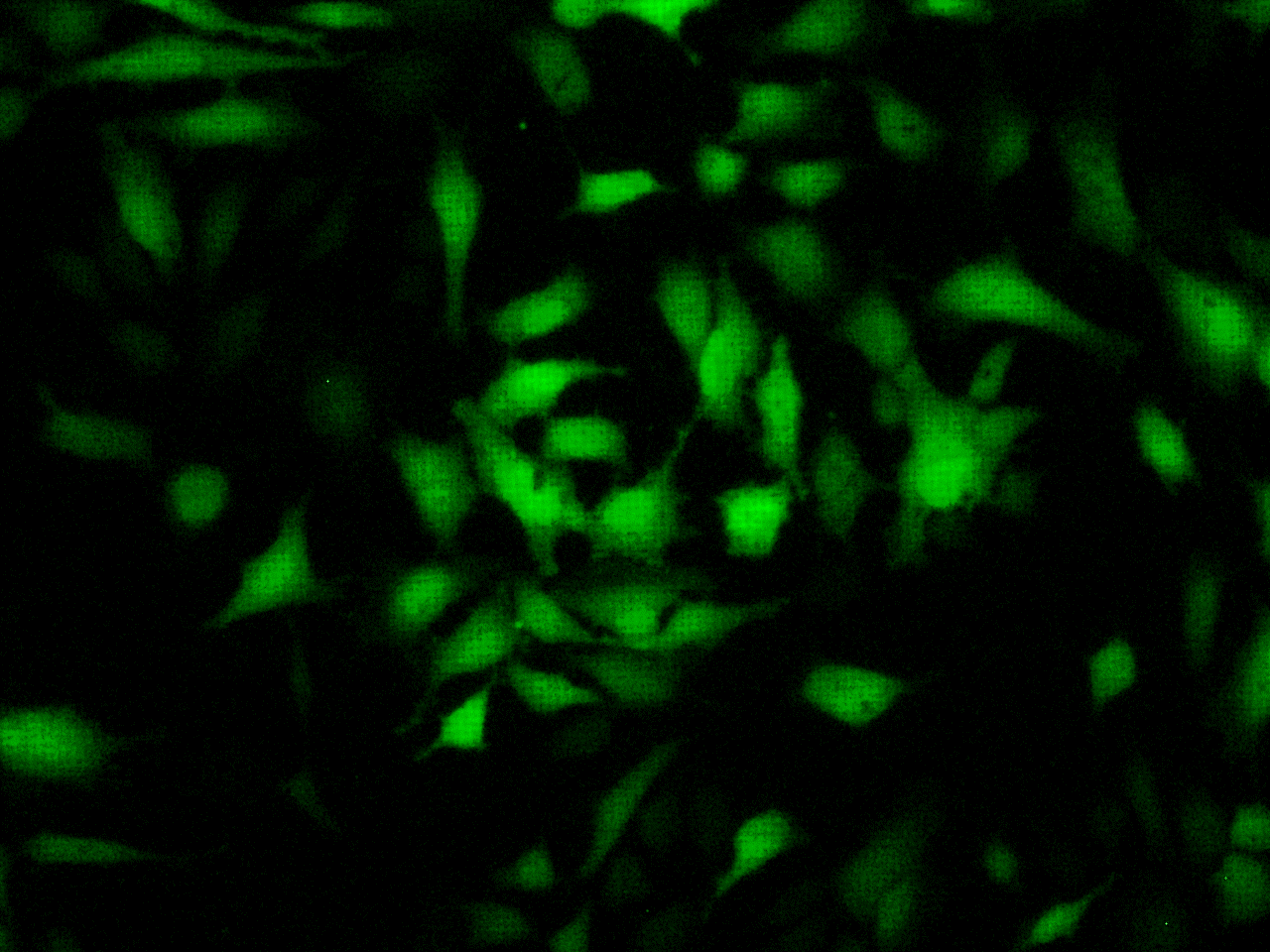





**Figure S9.** Cell counting technique. (A) The image of viable cells from Fig. 4B. (B) The image of porated cells from Fig. 4A. (C) The outlines of each viable cell from Fig. S9A. (D) An overlay of the viable cell outlines from Fig. S9C on top of the porated cells from Fig. S9B. There are no green cells without a white, viable outline, meaning that all porated cells are viable.
